# Theory of mind in utterance interpretation: the case from clinical pragmatics

**DOI:** 10.3389/fpsyg.2015.01286

**Published:** 2015-08-26

**Authors:** Louise Cummings

**Affiliations:** English, Culture and Media Studies, School of Arts and Humanities, Nottingham Trent UniversityNottingham, UK

**Keywords:** clinical pragmatics, theory of mind, utterance interpretation, pragmatic disorder, reasoning

## Abstract

The cognitive basis of utterance interpretation is an area that continues to provoke intense theoretical debate among pragmatists. That utterance interpretation involves *some* type of mind-reading or theory of mind (ToM) is indisputable. However, theorists are divided on the exact nature of this ToM-based mechanism. In this paper, it is argued that the only type of ToM-based mechanism that can adequately represent the cognitive basis of utterance interpretation is one which reflects the rational, intentional, holistic character of interpretation. Such a ToM-based mechanism is supported on conceptual and empirical grounds. Empirical support for this view derives from the study of children and adults with pragmatic disorders. Specifically, three types of clinical case are considered. In the first case, evidence is advanced which indicates that individuals with pragmatic disorders exhibit deficits in reasoning and the use of inferences. These deficits compromise the ability of children and adults with pragmatic disorders to comply with the *rational* dimension of utterance interpretation. In the second case, evidence is presented which suggests that subjects with pragmatic disorders struggle with the *intentional* dimension of utterance interpretation. This dimension extends beyond the recognition of communicative intentions to include the attribution of a range of cognitive and affective mental states that play a role in utterance interpretation. In the third case, evidence is presented that children and adults with pragmatic disorders struggle with the *holistic* character of utterance interpretation. This serves to distort the contexts in which utterances are processed for their implicated meanings. The paper concludes with some thoughts about the role of theorizing in relation to utterance interpretation.

## Introduction

Few post-Gricean pragmatists would deny the central role of mind-reading or theory of mind (ToM) in utterance interpretation. But what is altogether more contentious is the exact nature of ToM in the complex cognitive processes whereby speakers produce, and hearers interpret utterances. This paper presents a particular view of this ToM-based process that is not popular among pragmatists or cognitive scientists in general. But it is a view that is supported by evidence of how utterance interpretation is impaired in children and adults with a range of pragmatic disorders. The view in question depends on three main claims. The first of these claims is that utterance interpretation involves the full exercise of rationality. When language users produce and interpret utterances, they are not constrained to operate within a particular rational sub-domain that has been identified by some theorists as *communicative* rationality. Rather, they are exercising a rational capacity, the key attribute of which is that it transcends efforts to circumscribe it. The second claim is that utterance interpretation goes well beyond the recognition of intentions à la Grice. In fact, it involves the full gamut of cognitive and affective mental states as well as the attribution of these states in more or less complex ways to the minds of language users. The third claim relates to a feature of utterance interpretation which is almost never explicitly acknowledged by theorists. That feature concerns the holism of the knowledge that language users draw upon during their interpretation of utterances. Pragmatic accounts of utterance interpretation tend not to emphasize the essential unity of this knowledge, preferring instead to represent certain aspects of knowledge as relevant to the interpretation of utterances. The way in which such accounts misrepresent the holism of knowledge will be challenged in this article.

So, it will be argued that any ToM-based process that is to play a role in utterance interpretation must have a fully rational, intentional, holistic character in the particular senses outlined above. But such an understanding of utterance interpretation will not be acceptable to very many pragmatists and cognitive theorists. The contention that it is not possible to circumscribe the rational capacity that is exercised during utterance interpretation—at least if we are to end up with an *intelligible* account of this interpretation—will be unpalatable to cognitive theorists and pragmatists, many of whom have a substantial appetite for theory construction. However, it will be argued that although this proposal is unpalatable for many theorists in the area, it is an authentic representation of the rational character of utterance interpretation. The contention that the type of mental state attribution involved in utterance interpretation extends well beyond the recognition of communicative intentions will be troubling for those theorists (e.g., Sperber and Wilson) who believe that such attribution is the province of a highly specialized ToM module. And the contention that the knowledge and beliefs which we bring to utterance interpretation exist as a unified whole will be unsettling to any pragmatist who has ever talked about the beliefs and knowledge that are *relevant* to utterance interpretation (the implication, of course, is that there are other beliefs and knowledge that are *not relevant* to interpretation). Although each of these contentions will be disturbing to theorists who hold dear certain assumptions about utterance interpretation, these assumptions must be challenged if we are to begin to think in more productive ways about the cognitive basis of such interpretation. At least this will be our starting point for the following discussion of the nature and role of ToM in utterance interpretation.

That the three claims introduced above are valid statements about normal utterance interpretation will be demonstrated by examining impairments in the use and understanding of utterances in children and adults with pragmatic disorders. To the extent that utterance interpretation involves the exercise of a rational capacity, we might expect to find deficits in reasoning and the use of inferences in individuals with pragmatic disorders. Moreover, to the extent that this rational capacity has an open texture which evades circumscription, we may expect these deficits to be evident in domains beyond communication. The claim that utterance interpretation has an intentional character that goes beyond the recognition of intentions may also be verified on the basis of evidence obtained from clients with pragmatic disorders. We may expect to find deficits in the attribution of cognitive and affective mental states other than intentions in children and adults with these disorders. These states play a vital role in the interpretation of utterances, although this role is seldom acknowledged by pragmatists. The claim that the knowledge we bring to utterance interpretation exists as a unified whole also receives empirical validation from clients with pragmatic disorders. To the extent that the holism of this knowledge poses difficulties for clients with pragmatic disorders, we may expect them to process utterances within highly restricted contexts that are isolated from the wider body of knowledge to which they belong. It will be the aim of later sections to demonstrate that there is substantial empirical support for all three of these claims in clinical subjects. In the meantime, we consider the implications of these claims for the analysis of a standard communicative exchange of the type routinely examined in pragmatics.

## A standard communicative exchange

The analysis of a standard communicative exchange serves as a useful starting point for the following discussion. This analysis will emphasize the rational, intentional, holistic character of utterance interpretation. In doing so, it will force us to think differently—and, it is hoped, more critically—about the mainly modular proposals^1^ that have tended to dominate cognitive accounts of interpretation. Consider the exchange below between Mark and Jane:

1. Jane: Do you fancy going to Spain again this summer with my parents?
2. Mark: They didn't cope well with the heat last year.
3. Jane: Okay then. I'll ask Bill instead.

The apparent ease with which Jane recovers the implicature of Mark's utterance—Mark clearly does not wish to go to Spain in the summer with Jane's parents—belies the complexity of the cognitive processes that are integral to this exchange. In demonstration of these processes, we need to examine the subconscious steps which Jane must take in order to recover the implicature of Mark's utterance. Before Mark can establish the communicative intention that motivates Jane's utterance, he must first undertake a number of pragmatic developments of the logical form of Jane's utterance. He must establish the referent of the pronoun “you” and the period of time that Jane has in mind when she uses the expression “this summer.” He must also know the individuals that Jane is referring to through the use of the noun phrase “my parents.” Only when referents are obtained for the indexicals “you,” “this,” and “my” can Mark even be said to be in possession of the proposition that is expressed by Jane's utterance. But Mark's cognitive input to this exchange does not end with the pragmatically enriched proposition of Jane's utterance. For this proposition is then subject to further pragmatic processing. At least part of this processing leads Mark to the presupposition of the iterative expression “again” in Jane's utterance—the presupposition that Jane and Mark have been to Spain *before*. It is also this additional processing which enables Mark to see that Jane is doing more than merely posing a question in the above exchange. For Jane is simultaneously suggesting that Spain should be the destination of their next summer vacation and that her parents should be their traveling companions during this trip. It is only when this particular speech act is established that Mark can be said to have recognized the communicative intention that motivated Jane's original utterance.

From assigning referents to indexicals to establishing the illocutionary force of Jane's utterance, Mark must perform a range of complex cognitive processes in the above exchange. But he is not alone in this regard. Jane, too, must exercise similar cognitive processes if she is to succeed in making sense of Mark's contribution to this exchange. Jane must also undertake pragmatic developments of the logical form of Mark's utterance. She must establish that her parents are the referent of the pronoun “they” in Mark's utterance. She must also be able to establish a temporal referent for the expression “last year” in this utterance. Some concept narrowing is required to appreciate the meaning of “heat” in Mark's utterance. Jane must understand this term to mean the high temperatures in Spain rather than just a general state or quality of being hot. Even after she has arrived at the proposition which is expressed by Mark's utterance, Jane must engage in further pragmatic processing in order to obtain the implicature of his utterance. That implicature is calculable on the assumption that Mark is attempting to make a relevant contribution to the exchange notwithstanding his apparent failure to address the specific issue raised by Jane's direct question. That issue—Mark's willingness to undertake another trip to Spain in the company of Jane's parents—has significant implications for the social relationship that exists between Mark and Jane, particularly if Mark does not welcome the opportunity to spend more time with Jane's parents. Jane must use her knowledge of that relationship to decide that if Mark is going to decline her proposal to travel to Spain, he is most likely to do so indirectly by way of an implicature. The recognition of this particular implicature is signaled by Jane in her final utterance in the exchange when she states that she will present the same proposal to Bill instead.

It is through a complex interplay of cognitive processes that the utterances in the above exchange are meaningful to Mark and Jane. I have argued elsewhere that these processes take the form of a single, undifferentiated ToM-based mechanism which achieves the pragmatic enrichment of the logical form of an utterance and the recovery of implicatures proper (Cummings, 2014a). In order for Jane to establish the referent of the indexical “they” in Mark's utterance in the above exchange, she must attribute to him many of the same mental states that she will use to recover the implicature of his utterance. However, of more interest in the present discussion is not that a ToM-based mechanism is used in both the pre- and post-propositional processing of an utterance—we will take it as unproblematic that it is—but the exact nature of this ToM-based mechanism. Expanding on an earlier discussion in Cummings (2014b), it will be argued that this mechanism cannot be a cognitive module or other highly specialized inferential device and be an authentic representation of the cognitive processes involved in utterance interpretation, at least to the extent that the latter has the type of rational, intentional, holistic character proposed in this paper. To see that this is the case, we need only examine in more detail the cognitive processes which Mark and Jane must undertake in order to participate in the above exchange. These processes involve nothing short of a full-blown ToM of a type that lies well beyond the representational capacity of a cognitive module or other specialized inferential device. Empirical support for these processes is presented in later sections. In the remainder of this section, these processes are examined on their own terms.

That an unbounded rational capacity is exercised by Mark and Jane in the above exchange is a key component of the cognitive account of utterance interpretation proposed in this paper. If this capacity is a truly unbounded entity, as it is contended, then it should not be possible to place a limit on the rational considerations which come into play in the above exchange. However, if this capacity is a bounded construct which can be circumscribed and even modularized, then we must recognize a point at which a line can be drawn around the rational capacity that Mark and Jane are using in this exchange. That the former scenario best represents this rational capacity, both in relation to the calculation of implicatures and the primary pragmatic processes^2^ used to obtain the propositions of utterances, will now be demonstrated. Let us return to Mark's utterance in the above exchange. That utterance was taken to generate the implicature that Mark does not want to travel to Spain with Jane's parents in the summer. According to the standard pragmatic account of utterance interpretation, that implicature is arrived at by a process of reasoning which uses as its “premises” certain mutually held expectations about the rational conduct of (verbal communicative) behavior. These expectations require Mark to contribute only those utterances to the exchange which will have some relevance to, or salience for, his communicative partner Jane. Accordingly, any utterance that Mark contributes must relate to the topic of Jane's question (a summer trip to Spain) and to the specific proposal contained in that question (the proposal to travel to Spain with Jane's parents). Mark's actual utterance fulfills these criteria only to the extent that Jane is able to draw the following inferences:

1. The use of “they” in Mark's utterance refers to Jane's parents.
2. The use of “the heat” may be taken to mean the temperature in Spain.
3. The use of “last year” refers to the 12-month period prior to the speaking of Mark's utterance and, specifically, to the summer time within that period.

The primary pragmatic processes, which Jane must employ in order to achieve *reference assignment* and *lexical narrowing* in (1)–(3) above, are thus guided by her rational expectations of Mark in this communicative exchange. But unlike most pragmatic accounts, which would have Jane's communicative rationality end here, the inferences in (1)–(3) are themselves only intelligible to the extent that Jane is in possession of a number of other rational expectations. Several of these expectations relate to Mark's competence as a user of the English language. For example, Jane must have as a rational expectation that if Mark wants to refer to more than one person, he will know that he must use a plural pronoun in order to do so. Jane will also have a series of other rational expectations. For example, she will have an expectation that Mark will have a sound understanding of concepts such as time and physical properties like temperature, and that he can appropriately capture these concepts and properties in linguistic expressions such as “year” and “heat,” respectively. But Jane's rational expectations do not even end here. She will also have rational expectations about Mark's world knowledge such that she will expect him to know that Spain is a European country which has a warm climate. Jane will also expect Mark to know that it is this warm climate which makes Spain a popular destination for many tourists. In short, the inferences in (1)–(3) above presuppose an entire network of rational expectations which are not bounded in any way and cannot be circumscribed, as most pragmatic accounts of utterance interpretation would have it. What started out as a series of inferences, which were aimed at achieving reference assignment and lexical narrowing, quickly opened up into an array of rational expectations which were as complex as human thought itself.

The situation is no less complex when we consider the steps which Jane must take in order to obtain the implicature of Mark's utterance. To derive the implicature that Mark does not want to undertake a trip to Spain with Jane's parents, Jane must again engage in a process of reasoning which has certain rational expectations as its “premises.” These expectations lead Jane to search for the relevance of Mark's utterance as a response to her question. It is that search for the relevance or salience of Mark's utterance to Jane that leads her to draw the inference in (7) from the propositions in (4)–(6):

(4) If my parents cannot travel to Spain, then it will not be possible for Mark to undertake a trip to Spain with them.
(5) Mark has mentioned a factor—my parents' intolerance to heat—that may preclude them from undertaking a trip to Spain.
(6) Mark has mentioned this factor with a view to reducing the likelihood that such a trip will take place.
(7) Mark is implicating that he does not want to travel to Spain with my parents.

On most pragmatic accounts of utterance interpretation, the role of (communicative) rationality is limited to a number of rational expectations which secure the recovery of the implicature in (7). But it is not difficult to demonstrate that this cannot be the case. This implicature is derivable on the basis of certain rational expectations which serve to establish the relevance of Mark's utterance as a response to Jane's question. One of these expectations is that Mark is behaving as a cooperative communicator in his exchange with Jane. But this single expectation presupposes a range of other rational expectations that are equally important to the recovery of the implicature in (7). For example, Jane cannot have a rational expectation that Mark will be a cooperative communicator in the exchange in the absence of further rational expectations to the effect that Mark has intact linguistic competence and that he can employ this competence to communicate effectively in a range of contexts. The expectation that Mark has intact linguistic competence in turn presupposes other rational expectations about a range of conditions which Jane may reasonably assume apply to Mark. For example, she must have a rational expectation that Mark's language development has proceeded along normal lines, that his linguistic competence has not been impaired by disease or injury, and that Mark is not currently under the influence of chemical substances which may, temporarily at least, disrupt his competence. These additional rational expectations are integral to what it means for Jane to have a rational expectation that Mark is a cooperative communicator. As such, they are no less important to the recovery of the implicature in (7) than the expectation of cooperation which is routinely included in pragmatic accounts of utterance interpretation.

It is important to point out that the difference between the view of utterance interpretation proposed in this paper and that which is adopted by standard pragmatic accounts is not merely one of emphasis. For the network of rational expectations examined above plays a particularly critical role in utterance interpretation. It is through this network that Jane's expectation that Mark is a cooperative communicator is even an *intelligible* thought. Put quite simply, *no sense* can be made of Jane's expectation that Mark is behaving as a cooperative communicator in her exchange with him in the absence of this extensive network of rational expectations. For most pragmatists and cognitive theorists, this network is rarely even alluded to in their theoretical accounts of the cognitive basis of utterance interpretation. In fact, modular accounts of this interpretative process actively eschew the types of rational considerations which are emphasized in the present context. The modularization of any body of knowledge, should that knowledge be used to interpret utterances or perform other cognitive processes, can only proceed by excluding the prior rational expectations which, it was argued above, Jane must have in order for her to view Mark as a cooperative communicator. But in the absence of this prior rationality, this modularized knowledge is not even intelligible as an account of the cognitive basis of utterance interpretation. That so many present-day pragmatists and cognitive theorists subscribe to modular accounts of utterance interpretation, I have argued elsewhere, is symptomatic of an impulse to theorize about concepts such as rationality and meaning (Cummings, 2002a,b, 2005a,b, 2012a,b, 2014b). Those arguments will not be rehearsed here. Rather, we continue our examination of the cognitive basis of utterance interpretation.

Alongside an emphasis on the rational character of utterance interpretation, the view proposed in this paper also challenges us to think differently about the intentional character of this process. Of course, all post-Gricean pragmatic accounts of utterance interpretation acknowledge the central role of the recognition of intentions in this interpretation. It is only when a speaker's intention in producing an utterance is recognized that a hearer may even be said to have understood what the speaker means. However, communicative intentions, whilst important, are merely one type of mental state which hearers must attribute to the minds of speakers during utterance interpretation. Indeed, if anything, intentions are dependent upon a range of other cognitive and affective mental states which assume a primary role in the interpretation of utterances. To see this, let us return to the above exchange between Mark and Jane. In order for Jane to recover the implicature of Mark's utterance in this exchange, she must be able to establish the intention that motivated this utterance. Mark produced his utterance with the intention of inducing in Jane the belief that he, Mark, does not want to travel to Spain with Jane's parents. But this intention is a type of secondary mental state which is dependent upon other mental states. Although these other mental states are not intentions, they are no less important to the recovery of the implicature of Mark's utterance than the mental state of intention which is privileged in pragmatic accounts of utterance interpretation. An examination of the different mental states that Jane must attribute to Mark before she can even recognize the intention that motivates his utterance illustrates this point well.

In order to recover the implicature of Mark's utterance, Jane must attribute certain *knowledge* and *belief* states to Mark. These states include knowledge that Spain is a European country, and the belief that Jane's parents are intolerant to heat. Jane must also attribute to Mark a range of states based on *desire*. One such state is that Mark wants to maintain his pre-existing social relationship to Jane by declining her proposal to travel to Spain with her parents indirectly by way of an implicature. Jane must also attribute to Mark a desire to undertake foreign travel in order to present her proposal to him in the first place. Alongside knowledge, belief, and desire states, Jane must also attribute certain states of *ignorance* or lack of knowledge to Mark. For example, she must attribute a lack of knowledge of her summer travel plans to Mark in order to make her own verbal behavior—the revelation of those plans—a rational move in the above exchange. Jane's verbal behavior is also only rational to the extent that she is able to attribute to Mark a lack of knowledge of any forthcoming events that may coincide with, and preclude, a summer trip to Spain. Alongside cognitive mental states, a range of affective mental states are also integral to Jane's recovery of the implicature of Mark's utterance. Mark's smiling face and relaxed demeanor may lead Jane to attribute a state of *happiness* to him. To the extent that Jane wants Mark to accept her travel proposal, the attribution of this particular affective state to Mark may encourage Jane to present her proposal to him now rather than in 2 days' time, when she knows Mark must have a tooth extracted at the dentist. Jane may also attribute to Mark *disgust* of Spanish food, and a *fear* of flying, two affective mental states which she recognizes may incline Mark to reject her travel proposal.

It emerges that the full panoply of mental states—intentions, knowledge, beliefs, desires, ignorance, happiness, disgust, and fear—may be attributed to the mind of a speaker during the recovery of an implicature. It also emerges that intentions hold no special logical position within this wider set of mental states, notwithstanding their dominance in pragmatic accounts of utterance interpretation. The combination of these factors leads one to doubt whether any cognitive module which is specialized to process intentional data could even begin to represent the mental states that are involved in utterance interpretation. Like rational expectations before them, mental states exist not as isolable units, but as part of a larger network of intentional phenomena. Indeed, it is on account of this wider network that intentions and other states are even *intelligible* mental phenomena. It was demonstrated above that Jane is not simply attributing an intention to Mark when she interprets his utterance as a rejection of her proposal to travel to Spain. If anything, that intention was a type of secondary mental state that was only attributed to Mark after Jane had already attributed a range of other cognitive and affective mental states to him. That these other states are also instrumental to the recovery of Mark's implicature has implications for the type of cognitive structure which can play a role in utterance interpretation. Specifically, that structure cannot be a cognitive module that is specialized for the recognition of intentions, as most pragmatists would have it. In fact, any type of cognitive module serves only to exclude the very intentional phenomena that make the recognition of intentions during utterance interpretation intelligible. A quite different form of description is needed. Some thoughts about what that description may involve are addressed subsequently.

We turn to the final feature of the alternative view of utterance interpretation proposed in this paper. That feature is the holistic character of interpretation. To some extent, this feature has already been addressed. It has been argued that the rational expectations and intentional phenomena which are integral to utterance interpretation are not isolable in any sense but exist as a unified whole. This same holism applies to the knowledge which speakers and hearers bring to utterance interpretation. That knowledge is variously captured by pragmatists in expressions such as “background knowledge,” “mutual knowledge,” “shared knowledge,” and “world knowledge.” These expressions reflect the fact that speakers and hearers use knowledge not only of each other but also of states of affairs in the world during their interpretation of utterances. This was evident in Jane's exchange with Mark, where Jane used knowledge of Mark's mental states as well as her knowledge of people, places, and events in the world to derive the implicature that Mark does not want to travel to Spain in the summer with her parents. For most pragmatists, only certain aspects of Jane's knowledge are relevant to her interpretation of Mark's utterance in this exchange. So, while her knowledge that Spain is a European country may be judged to be relevant to her interpretation of Mark's utterance, her knowledge that Spain has had three recessions in the last 5 years may not be considered to be relevant. Also for most pragmatists, the former knowledge can be circumscribed within a cognitive module or other specialized inferential device, while the latter knowledge can be disregarded as somehow irrelevant to Jane's interpretative task. For the sake of argument, let us assume that this account of the knowledge that is used in utterance interpretation is not just possible but is obtained in the particular case of Jane's exchange with Mark. What would such a body of knowledge look like?

The answer to this question is that we do not have the first idea what such a body of knowledge would look like. In fact, we must concede the complete *unintelligibility* of this knowledge. To understand why, we need only examine further the knowledge that Jane brings to her interpretation of Mark's utterance in the above exchange. It was suggested that this knowledge might contain the proposition that Spain is a European country. This proposition may even be represented within a cognitive module that is specialized for utterance interpretation. And for most pragmatists, the matter ends here. But it is not difficult to demonstrate that this single proposition depends on other propositions for its own intelligibility. For example, the proposition that Spain is a European country presupposes propositions to the effect that Europe is a continent and that Spain is one of several nations in the continent of Europe. Let us assume that both of these propositions are also represented within the cognitive module that Jane uses to interpret Mark's utterance. Surely now we can draw a line around the knowledge that must be permitted entry to the module. But the matter does not end here either. For the module must also contain knowledge to the effect that Spain and other European nations have their own cultures and languages, that some of these languages (e.g., Portuguese) are also spoken in South American countries and that bull fighting is a cultural tradition in Spain. The point that is demonstrated by means of this example is that there is no stage at which we can throw a net around the knowledge that Jane uses during utterance interpretation and then claim this knowledge to be complete. *A fortiori*, Jane's knowledge cannot be fully circumscribed within a cognitive module, even one which is specialized for utterance interpretation.

The problem that the holism of knowledge poses for pragmatists and cognitive theorists is that it is not possible to circumscribe the knowledge that we bring to utterance interpretation *and* arrive at an *intelligible* account of that knowledge. Regardless of where we think we can draw a boundary around the knowledge that is relevant to utterance interpretation, it can be readily demonstrated that it is only possible to make sense of this circumscribed knowledge by using knowledge that lies outside of the boundary. This boundary typically takes the form of an encapsulated cognitive module or a series of such modules, each of which is specialized to perform a particular function. This modular account has a certain appeal to pragmatists. It appears to be complete in the sense that a cognitive module contains all the knowledge that is relevant to utterance interpretation. It also appears to embody cognitive efficiencies in that the need for extensive searches of background knowledge is obviated when knowledge that is relevant to utterance interpretation is brought together in a specialized cognitive module. But this completeness and efficiency are more illusory than real. For what we have produced is not a complete account of utterance interpretation but an unintelligible account, which lacks a prior concept of knowledge with which to make sense of the circumscribed contents of a cognitive module. The dilemma that confronts pragmatists is the same dilemma that confronts any cognitive theorist who believes it is possible to produce a complete theoretical account of concepts such as meaning, rationality, and knowledge. Such an account appears to achieve the completeness of a theory. However, it only does so by eschewing the very rational and epistemic concepts that make that account intelligible.

Thus far, the discussion has addressed a number of conceptual issues relating to the cognitive basis of utterance interpretation. It has been important to reflect on these issues for at least two reasons. First, they have encouraged us to take a critical stance toward dominant (modular) accounts of utterance interpretation. Second, these issues have also encouraged us to think about what an alternative account of utterance interpretation might look like, especially one that is construed along the rational, intentional, holistic lines proposed in this paper. Having addressed these conceptual issues, we are now in a position to consider if there is any empirical support for this alternative view of utterance interpretation. That support, it will be argued, is to be found in a range of clinical disorders. Specifically, children and adults with pragmatic disorders exhibit problems in the use and understanding of utterances which are consistent with the alternative view of utterance interpretation that has been outlined above. It is to an examination of these disorders, and their implications for an account of utterance interpretation, that we now turn.

## Empirical support from pragmatic disorders

The view of utterance interpretation proposed in this paper receives substantial empirical support from a range of pragmatic disorders. That view is expressed in a claim to the effect that utterance interpretation has a rational, intentional, holistic character. As a means of validating this claim, three types of clinical case will be considered in this section. To the extent that utterance interpretation involves the exercise of a fully unconstrained rational capacity, and not some narrowly defined communicative rationality, the first of these clinical cases presents evidence of the presence of deficits in reasoning and the use of inferences in domains beyond communication in subjects with pragmatic disorders. It was also argued above that intentions represent a mere subset of the cognitive and affective mental states that must be attributed to the minds of speakers during utterance interpretation. To the extent that this is the case, we may expect to find evidence of deficits in the attribution of a range of mental states beyond those of intention in children and adults with pragmatic disorders. It was also argued above that any account of the cognitive basis of utterance interpretation must be able to represent the holism of knowledge. To the extent that the knowledge we bring to utterance interpretation exists as a unified whole, we may expect to find evidence of a tendency in children and adults with pragmatic disorders to process utterances within restricted or limited contexts. These contexts may be expected to privilege certain (dominant) meanings of words and utterances and limit the extent to which hearers seek alternative (non-dominant) meanings. Having examined the empirical support which exists for this view of utterance interpretation, the paper concludes with some thoughts about its implications for *theories* of utterance interpretation.

### Deficits in reasoning and inference

There is now extensive evidence of deficits in a range of inferences related to utterance interpretation in clients with pragmatic disorders^3^. Deficits in reasoning and inference have been reported in children with specific language impairment (SLI) and primary pragmatic difficulties (Botting and Adams, 2005; Adams et al., 2009), high-functioning children with autism (Dennis et al., 2001), children with attention deficit hyperactivity disorder (McInnes et al., 2003; Berthiaume et al., 2010) and hydrocephalus (Dennis and Barnes, 1993; Barnes and Dennis, 1998), and in pediatric traumatic brain injury (Dennis and Barnes, 2001; Moran and Gillon, 2004). Deficits in inferential aspects of utterance interpretation have also been reported in adult-onset conditions including schizophrenia (Corcoran, 2003), multiple sclerosis (Laakso et al., 2000), and right-hemisphere damage (RHD) (Tompkins et al., 2000, 2001, 2009; Lehman-Blake and Tompkins, 2001). These studies certainly support the claim that there is disruption to inferences which play a role in utterance interpretation. But this claim does not go far enough for our present purposes. In order to support the contention that utterance interpretation involves the exercise of a fully unconstrained rational capacity, we must also be able to identify deficits in reasoning and inference in non-communicative domains. In effect, we must be able to give an affirmative answer to the question: Is there any evidence that children and adults with pragmatic disorders also experience deficits in reasoning and inference in areas other than utterance interpretation? These deficits include impairments across a range of inference types *and* cognitive domains, and not just those inferences which are associated with utterance interpretation. It will be argued that evidence to this effect can be readily presented for a number of the pragmatic disorders introduced above.

It is not difficult to demonstrate the existence of impairments in a range of inference types in clients with pragmatic disorders. The breadth of these inferential impairments across domains provides support for the view that an unconstrained rational capacity is exercised during utterance interpretation. Children with SLI exhibit poorer deductive reasoning (Newton et al., 2010) and analogical reasoning (Leroy et al., 2012, 2014) than normally developing children. Individuals with autism spectrum disorder (ASD) exhibit deficits in analogical reasoning particularly about non-living items (Krawczyk et al., 2014) and defeasible conditional reasoning (Pijnacker et al., 2009). Adolescents with moderate to severe traumatic brain injury exhibit impairments in analogical reasoning ability (Krawczyk et al., 2010). A broad range of inferential deficits also exists in adult-onset conditions. Adults with schizophrenia display impaired probabilistic inference (Averback et al., 2011), transitive inference (Titone et al., 2004), associative inference (Armstrong et al., 2012), analogical reasoning (Simpson and Done, 2004), inductive reasoning (Corcoran, 2003), and deductive reasoning (Mirian et al., 2011). In a study of patients with acute aphasia, non-linguistic abstract reasoning was the only cognitive domain not to show improvement in the first year after stroke (El Hachioui et al., 2014). Adults with penetrating head injuries and focal lesions to the parietal cortex display deficits in transitive reasoning (Waechter et al., 2013). Adults with a range of dementias also exhibit deficits in reasoning. Yoshiura et al. (2011) found evidence of deterioration of abstract reasoning ability in individuals with Alzheimer's disease and amnestic mild cognitive impairment. Vartanian et al. (2009) reported that patients with frontal variant frontotemporal dementia (FTD) display impairments when engaging in transitive reasoning about familiar spatial environments.

These studies clearly demonstrate that individuals with pragmatic disorders experience an array of inferential deficits. The fact that these deficits also occur across domains such as reasoning about concrete and abstract entities, about living and non-living items and during language processing and visuospatial cognition suggests that there is disruption to a central rational capacity in individuals with pragmatic disorders rather than impairment of a specialized communicative rationality. Just such a rational capacity is posited in the view of utterance interpretation proposed in this paper. That the exercise of a fully unconstrained rationality is at work in utterance interpretation is now supported on conceptual *and* empirical grounds. On conceptual grounds, it was shown that the rational expectations which make communication possible are only intelligible to the extent that there exist other rational expectations which are as wide-ranging as human thought itself. It is simply not possible to circumscribe or modularize the rational expectations, thoughts, and concepts that play a role in utterance interpretation. This conceptual argument in favor of an unconstrained rational capacity receives empirical support from the study of pragmatic disorders. It was argued that children and adults with pragmatic disorders do not merely display impairments in the use of language-based inferences. Rather, inferential impairments in these subjects cut across cognitive domains and types of reasoning. The latter findings suggest that a central rational capacity is disrupted in individuals with pragmatic disorders, and not some rational sub-domain that is specialized for communication. But we must go further than the demonstration of an unconstrained rational capacity if the present view of utterance interpretation is to be upheld. For that view also makes specific claims about the intentional character of this process. It is to an examination of the empirical support for these claims that we now turn.

### Deficits in mental state attribution

It was argued above that post-Gricean pragmatic accounts of utterance interpretation routinely acknowledge the central role of the recognition of intentions in communication. Many of these accounts also argue for the existence of an inferential device or cognitive module that has become specialized to the task of intention recognition (e.g., Sperber and Wilson's relevance theory). That the recognition of intentions is integral to communication is one of the few indisputable facts of utterance interpretation. But what is often overlooked is that the type of mental state attribution involved in utterance interpretation extends more widely than the attribution of communicative intentions to the minds of speakers. In fact, the interpretation of any linguistic utterance involves the attribution of the full range of cognitive and affective mental states to the minds of other communicators. To the extent that this wide-ranging intentional capacity is implicated in utterance interpretation, it should be possible to find evidence of deficits in the attribution of mental states other than intentions in children and adults with pragmatic disorders. These states include cognitive mental states like knowledge, belief, and pretense and affective mental states such as happiness, fear, and anger. To the extent that evidence of this kind is forthcoming, it may be used to support two claims. The first of these claims is that there is no limit on the type of mental states that may play a role in utterance interpretation and that may be disrupted when interpretation is impaired. The second claim is that it makes no sense to talk about a cognitive module that is *specialized* to undertake the recognition of intentions when such a device would require nothing less than the modularization of the whole of human thought about the minds and behavior of other people. The necessary general nature of this cognitive module precludes any such specialization.

That the recognition of communicative intentions is impaired in individuals with pragmatic disorders has been demonstrated in a number of studies. For the most part, these studies reveal a failure on the part of subjects to recover the implicatures of utterances or establish the illocutionary force of speech acts. In this way, children with SLI have been found to have difficulty deriving scalar implicatures (Katsos et al., 2011), while children with pragmatic language impairment (PLI) perform significantly more poorly than those with SLI on questions targeting implicature (Ryder et al., 2008). Pragmatic impairments in schizophrenia are known to compromise the comprehension and recognition of speech acts, maxims and implicatures (Tényi et al., 2002; Mazza et al., 2008). McNamara et al. (2010) reported that patients with Parkinson's disease are less likely than control subjects to activate indirect meanings of implicatures. The interpretation of implicatures is also impaired in adults with RHD (Kasher et al., 1999). These studies support the claim that communicative intentions are a problematic mental state category for children and adults with pragmatic disorders. But then so, too, are a range of other cognitive and affective mental states. Children with autism and Asperger syndrome (AS) have been found to refer predominantly to desire and make few references to thought and belief in their use of assertive speech acts (Ziatas et al., 2003). Normally developing children in the same study used a higher proportion of references to thought and belief. Pretense is a problematic mental state for young children and adolescents with autism (Bigham, 2008; Morsanyi and Handley, 2012). Affective mental states are also impaired in autism. Philip et al. (2010) found deficits in the recognition of basic emotions (happiness, sadness, anger, disgust, and fear) across facial, body movement and vocal stimuli in adults with ASD.

Beyond autism, cognitive and affective mental states are also impaired in a range of other clinical populations with significant deficits of utterance interpretation. Children and adults who have genetic syndromes with or without intellectual disability exhibit difficulties with a range of mental state categories. Porter et al. (2008) found a specific deficit in understanding false belief in the subjects with Williams syndrome in their study. Ho et al. (2012) found that individuals with velo-cardio-facial syndrome show impairments in the attribution of complex mental states to abstract visual stimuli. The attribution of a range of mental states is also disrupted in adult-onset conditions. Cognitive and affective ToM is impaired in patients with paranoid schizophrenia (Montag et al., 2011). Individuals with a high level of negative symptoms of schizophrenia have been found to display selective impairment in their ability to attribute affective mental states (Shamay-Tsoory et al., 2007). There are impairments of cognitive and affective ToM in individuals with sematic dementia, with awareness of affective but not cognitive ToM persisting into the moderate stage of the disease (Duval et al., 2012). Patients with FTD are impaired relative to controls in the recognition of the emotions of anger, fear, disgust, and happiness through facial features (Oliver et al., 2014). These patients also mislabeled negative facial expressions as happy more often than controls, a finding that suggested a deficit in the representation of positive affect in FTD. Henry et al. (2006) found that the recognition of basic emotions (e.g., disgust, anger) and the capacity for mental state attribution was significantly reduced in 16 adults with traumatic brain injury relative to controls.

It is clear from these studies that the meta-representational deficit in clients with pragmatic disorders extends well beyond the recognition of intentions. What can we conclude from this finding? The only possible conclusion is that it makes no sense to talk about a cognitive module that is specialized for the recognition of intentions, or even just *communicative* intentions, when the type of meta-representational capacity involved in utterance interpretation extends into every aspect of our thinking about the thoughts and behavior of other people. Such a general cognitive capacity cannot be represented by a cognitive module, even a module that is constructed along the broadest possible lines, *and* be intelligible in the absence of a range of intentional data that lie outside of the module. In the end, the intentional character of utterance interpretation comes to mean much more than the recognition of communicative intentions. For these intentions only even make sense within a complex network of other cognitive and affective mental states which are as pervasive as human thought itself. What makes it seem that these intentions can be removed from this network and represented in their entirety within a cognitive module is the assumption that it is possible to develop a *theory* of these mental phenomena. That assumption will be critically evaluated in the final section below. In the meantime, we turn to the third and last feature of utterance interpretation that is proposed in this paper. That feature concerns the holism of the knowledge that we bring to utterance interpretation.

### Deficits in background knowledge

It is important to begin the discussion of this final feature of utterance interpretation with a note of caution. The deficits in background knowledge that we will address in this section should not be taken to mean that individuals with pragmatic disorders do not know that France is a European country, that potatoes are a type of vegetable and that fish live in water. On the contrary, most children and adults with pragmatic disorders know all these things and more. Rather, what is being claimed here is that individuals with pragmatic disorders tend to interpret utterances within limited or restricted epistemic contexts. The key feature of these contexts is that they circumscribe the knowledge that hearers could potentially use to interpret utterances. While this tendency may simply reflect the wider processing limitations of subjects with pragmatic disorders—a context of just a few propositions is easier to retain in memory, etc.—its effect on utterance interpretation can be devastating. For example, the implicature that a hearer may derive from an utterance in a small, restricted context may not be the implicature that the speaker intended to convey. Also, it may not be possible to overturn or defeat an implicature that is immune to changes within the wider network of knowledge that attends utterance interpretation. Such a hearer may persist in upholding a particular implicature of an utterance or the dominant meaning of a word when it is clear from the wider context that such interpretations are erroneous. In this section, we will be concerned to establish if such a pattern of misinterpretation actually exists among children and adults with pragmatic disorders. To the extent that it does, we will have some empirical support for the claim that any account of utterance interpretation must succeed in representing the essential holism of knowledge.

Children and adults with pragmatic disorders often experience significant difficulties in the processing of context. These difficulties are typically documented during tasks that require the resolution of ambiguities based on linguistic context^4^. Jolliffe and Baron-Cohen (1999) reported that normally intelligent adults with autism or Asperger's syndrome are less able than normal controls to use context to interpret lexically or syntactically ambiguous sentences that are presented auditorily. Using a lexical ambiguity resolution task, Norbury (2005) demonstrated that children with language impairment and ASD plus language impairment do not use context as efficiently as their language intact peers to suppress irrelevant meanings. Difficulties suppressing contextually irrelevant meanings have also been reported in children with hydrocephalus (Barnes et al., 2004). Andreou et al. (2009) examined sentence context effects in homonym meaning activation in patients with schizophrenia. Unlike control subjects, who exhibited a pattern of selective target facilitation following the presentation of sentences which biased either the first or second meaning of equibiased homonyms, no significant target facilitation was observed in the patients with schizophrenia in this study. Grindrod and Baum (2003) examined the ability of subjects with right-hemisphere damage (RHD) and left-hemisphere damage (LHD) and nonfluent aphasia to use local sentence context information to resolve lexically ambiguous words. Subjects with nonfluent aphasia activated both meanings of ambiguous words regardless of context at a short interstimulus interval and neither meaning at a long interstimulus interval. The only contextually appropriate meanings to be activated in the subjects with RHD occurred in second-meaning biased contexts at a long interstimulus interval. Grindrod and Baum concluded that LHD and RHD lead to deficits in using local context information to complete ambiguity resolution.

Aside from ambiguity resolution, there is also extensive evidence of the failure of subjects with pragmatic disorders to use context appropriately during the processing of utterances for their implicatures. Ryder et al. (2008) examined the ability of two groups of typically developing children and 27 children with SLI to use context to generate implicatures in response to questions. Nine of the 27 children with SLI were pragmatically impaired. Only when an answer to a question was provided by pictorial context did the children with SLI perform similarly to their peers in the use of context to generate implicatures. However, children with PLI performed significantly more poorly than the rest of the SLI group on questions that required implicatures, leading the authors to conclude that these children have particular difficulty in integrating contextual information. Loukusa et al. (2007) examined the answers given by children with AS or high-functioning autism (HFA) to contextually demanding questions. Analyses of the answers given by these children revealed that they had all tried to use contextual information, albeit that they had done so incorrectly. The examination of a category of error not produced by the normally developing children in the study indicated that the children with AS or HFA continued to process questions even after a contextually relevant answer had been given. Titone et al. (2002) found that patients with schizophrenia showed reduced priming for literally plausible idioms (e.g., kick the bucket) but intact priming for literally implausible idioms (e.g., be on cloud nine) compared with control subjects. These authors concluded that patients with schizophrenia can make normal use of context only when conditions (e.g., the implausibility of certain idiomatic meanings) reduce the need for controlled processing.

By way of summary, let us reflect on the significance of these empirical findings for the holism of knowledge during utterance interpretation. These studies demonstrate that processing limitations in individuals with pragmatic disorders lead to the interpretation of utterances in highly restricted contexts. Within these contexts, utterances and lexically ambiguous words are frequently misinterpreted, as individuals with pragmatic disorders are unable to revise their understanding of language to reflect wider contextual information. In effect, pragmatic disorders directly disrupt the holism of the knowledge that we bring to utterance interpretation. The processing limitations of children and adults with these disorders forces them to view this background knowledge as containing isolable elements that can exist apart from other contextual information. The erroneous interpretations arrived at by the subjects in the above clinical studies is a clear demonstration of what can go wrong when such a view of this knowledge exists. What appears to be a restricted, self-contained context of background knowledge is in fact a complex informational nexus that is co-extensive with human thought itself. No component or element of this nexus can be separated from any other component or element *and* be an intelligible representation of the context that attends the interpretation of utterances. What makes it seem otherwise is a strong impulse to theorize about the cognitive basis of utterance interpretation. This impulse can now be seen to distort the holistic character of utterance interpretation in much the same way that it distorted the rational and intentional character of this cognitive process. In the next section, we consider the only possible route through this theoretical impasse.

## The way forward

Throughout this discussion, the urge to theorize about the cognitive basis of utterance interpretation has been cast in the role of villain. It is now time to examine that urge directly, and explain why it has such disastrous consequences when utterance interpretation is at issue. Theories of a whole range of phenomena abound in science and elsewhere. We do not think it strange if physicists develop theories of the gravitational forces between the earth and the moon. In fact, we would be surprised if we discovered that such theories did not exist. Theories explain and predict events and behavior in the world, and our ability to make sense of our environment would be significantly diminished without them. Theories strive for completeness in that they must account for all the data within a particular domain. And as any scientist will tell you, a theory that cannot account for all the data in an area will be very short lived indeed. But when we turn to utterance interpretation, the idea that it is possible to develop a theory of this phenomenon is quite a different proposition altogether. The completeness aspired to by theorists in other areas of inquiry is decidedly destructive when we turn to a rational, intentional, holistic phenomenon like utterance interpretation. For here the focus of our theoretical efforts are concepts such as rationality and intentionality which, as we have seen, are nothing short of human thought itself. The physicist who develops a theory, even a fully complete theory, of the gravitational forces between the earth and the moon still has a set of rational concepts with which to make sense of that theory. The pragmatist who develops a theory of the cognitive basis of utterance interpretation must arrive at a fully complete account of rationality and intentionality. But in the absence of rational concepts outside of this theory, the pragmatist's theoretical enterprise lacks the *intelligibility* of the physicist's enterprise.

This view of the unintelligibility of *theories* of rationality and intentionality derives from the philosophical insights of Hilary Putnam (e.g., Putnam, 1988, 1994, 1995)^5^. For many years, Putnam has railed against a certain way of doing philosophy which can make it seem that the only way in which we can make progress on concepts such as truth, meaning, and rationality is to construct theories of these concepts. Such theories, Putnam argues, only *appear* intelligible on the assumption that we can occupy a metaphysical standpoint. From this standpoint, it seems that we can survey human thinking in its entirety without in turn presupposing the rational concepts which make that thinking intelligible. But, to the extent that this standpoint is devoid of rational concepts (how else are we to achieve the *completeness* of a *theory* of human rationality?), what we end up with is not a complete account of rationality or meaning but an *unintelligible* account. In fact, in the absence of prior rational concepts, such a standpoint is a “we know not what.” In effect, the pragmatist who believes it is possible to generate a theory of utterance interpretation is in the same position as the metaphysical realist who has theoretical aspirations in relation to philosophical concepts. The pragmatist believes it is possible to capture the rational, intentional, holistic character of utterance interpretation within a cognitive scientific theory. This theory might be constructed around a cognitive module, or a series of such modules, or some other inferential device. However, if the discussion of the preceding sections has demonstrated anything, it is that such a theory is nothing less than an account of human thought. But at that point, what we have is not a complete account of utterance interpretation but an unintelligible account. Like the metaphysical realist, the pragmatist has not succeeded in producing an account that we can recognize, let alone make sense of.

Putnam's challenge is to the theoretical impulse which makes it seem that a cognitive scientific theory of utterance interpretation is possible and intelligible. But he is not alone in finding the entire research program that this impulse represents flawed and incoherent. John Searle exhibits the same concerns in relation to cognitivism, which is the view that the brain is a digital computer. For Searle, the proposal that the mechanisms by which brain processes produce cognition are supposed to be computational, and that by specifying programs we have specified the causes of cognition is no type of coherent explanation at all:

“I used to believe that as a causal account, the cognitivist's theory was at least false, but I now am having difficulty in formulating a version of it that is *coherent* even to the point where it could be an empirical thesis at all” (Searle, 1992: 215; italics added).

This lack of coherence arises, according to Searle, because the cognitivist denies that the characterization of a process as computational is an observer-relative characterization. A conscious agent must assign a computational interpretation to a pattern of physical events. In the absence of this agent, all we have are neurobiological processes which are not causal explanations of anything:

“The point is not that the claim “The brain is a digital computer” is simply false. Rather, it does not get up to the level of falsehood. *It does not have a clear sense*. The question “Is the brain a digital computer?” is ill defined. If it asks, “Can we assign a computational interpretation to the brain?” the answer is trivially yes, because we can assign a computational interpretation to anything. If it asks, “Are brain processes intrinsically computational?” the answer is trivially no, because nothing is intrinsically computational, except of course conscious agents intentionally going through computations” (Searle, 1992: 225; italics added).

A complete cognitive scientific theory (Putnam) and an intrinsically computational brain process (Searle) are just different manifestations of the same aberrant impulse in cognitive science. That impulse is to deny the existence of any rationality *outside* of the theory or the causal explanation. Yet, without this prior rationality we lack the very concepts that are needed to make sense of the cognitive scientist's theories and causal explanations. That such theories and explanations are unintelligible by their own standards is a clear sign that all is not well in the cognitive enquiry which has brought us to this point.

So, if a *theory* of utterance interpretation is not just a bad idea, but an unintelligible one, then what is the alternative? Can we afford to take seriously the proposal to reject modular and other theoretical accounts of utterance interpretation? And if we reject these accounts, is there anything that we can intelligibly say about utterance interpretation? For Searle, the way forward lies in an inversion of the order of our cognitive scientific explanations so that we get a different account of cause-and-effect relations in these explanations. Our psychological explanations are misguided when they posit deep unconscious mental causes of desired effects such as perceptual judgments or grammatical sentences. Rather, what appear to be mental causes of patterns in perception or language are actually the judgments of a conscious agent who is outside the perceptual and linguistic systems:

“The inversion radically alters the ontology of cognitive science explanation by *eliminating a whole level of deep unconscious psychological causes*. The normative element that was supposed to be *inside the system* in virtue of its psychological content now comes back in when *a conscious agent outside the mechanism makes judgments about its functioning*” (Searle, 1992: 237; italics in original).

Applied to utterance interpretation, it is Searle's claim that we are mistaken when we posit modular processes that somehow stand behind, and give a causal explanation of, our understanding of utterances. There are “brute physical mechanisms” in our brain which cause and sustain conscious thoughts, experiences, actions, and memories. But that is all there is. There is no level of deep unconscious mental processes which give a causal explanation of these thoughts and experiences. There is no intrinsic intentionality in any of the mechanisms we are attempting to explain, only in the conscious agents who are making judgments of these mechanisms:

“The elimination of the deep unconscious level marks two major changes: It gets rid of a whole level of psychological causation and it shifts the normative component out of the mechanism to the eye of the beholder of the mechanism” (Searle, 1992: 238).

Searle's dissatisfaction with cognitive scientific accounts of mind is matched by Putnam's concerns that the entire cognitive scientific venture has led us into unintelligibility. Putnam, too, seeks a different type of explanation, one in which the “eye of the beholder” can tell us something about normative concepts such as meaning and rationality in a way that cognitive scientific theories have failed to. Importantly, the eye of the beholder is *not* a “God's Eye point of view” or metaphysical standpoint, from which it is assumed we can survey the whole of rational thought without, in turn, presupposing rational concepts. It is the assumption of this standpoint which makes it seem that it is possible to generate complete cognitive scientific theories in the same way that it is possible to generate complete scientific theories of physical phenomena in the world. Like Searle's “rediscovery of the mind,” Putnam believes it is possible to recover an intelligible position in the philosophy of mind. It is part of Putnam's own attempt at recovery—what he has described as common-sense realism and a “deliberate” or “second naivete” about conception—that he would have us take seriously the teachings of Wittgenstein. This requires that we engage in a process of description, the aim of which is an accurate characterization of the consequences that a particular picture, and the concepts inherent in it, has for its user. In his *Lectures and Conversations on Aesthetics, Psychology, and Religious Belief*, Wittgenstein (1966) describes the considerations that are subsumed within this type of description:

“God's eye sees everything”—I want to say of this that it uses a picture.
I don't want to belittle…the person who says it…
We associate a particular use with a picture…
What conclusions are you going to draw?…Are eyebrows going to be talked of, in connection with the Eye of God?…
If I say he used a picture, I don't want to say anything he himself wouldn't say. I want to say he draws these conclusions.
Isn't it as important as anything else, what picture he does use?…
The whole *weight* may be in the picture…When I say he's using a picture, I am merely making a *grammatical* remark: [What I say] can only be verified by the consequences he does or does not draw…
All I wished to characterize was the consequences he wished to draw. If I wished to say anything more I was merely being philosophically arrogant (pp. 71–72).

The most outstanding feature of this descriptive process is the restrictions placed on the extent of the description. Wittgenstein (1966) doesn't want to say anything he—the user of the picture—himself wouldn't say. Indeed, to say more is “being philosophically arrogant.” In fact, to say more is to proceed to philosophize in the manner urged by the metaphysical spirit, a manner in which we describe the application of a picture through an understanding of that same picture in isolation from its applications. Under the influence of the metaphysical spirit, we inevitably go forward by erecting standards about what must be the case in order for our thoughts to represent or refer to reality. These standards can make it seem that there must be something which stands behind thoughts and which makes it possible for them to represent the world. This “something” is unconscious mental processes which, it is claimed, provide a causal explanation of our conscious thoughts. It is these processes which the cognitive scientist aims to give an account of in his or her theories. But these processes are nothing but an illusion which arises, Putnam contends, when we attempt to characterize normative concepts like meaning apart from the wider nexus of rational concepts that is their home. As Searle (1992) remarks “deep unconscious rules satisfy our urge for meaning” (246). However, we are looking in the wrong place if we think an account of meaning, rationality, and other normative concepts lies anywhere other than conscious agents who use utterances to mean such and such.

Cognitive scientific accounts of utterance interpretation also appear to “satisfy our urge for meaning.” Unconscious modular processes in particular appeal to our sense that “if the input to the system is meaningful and the output is meaningful, then all the processes in between must be meaningful as well” (Searle, 1992: 246). But there is no intentionality in the utterance interpretation system, only in the conscious agents who attempt to characterize that system. And it is from these agents that serious philosophical work on concepts such as meaning and rationality must proceed. In unpicking the complexity of these concepts in Section A Standard Communicative Exchange, we employed a form of description that opened up the rich interconnections between them. Such was the extent of our mining of these interconnections that it very quickly became apparent that we could not make any sense of a concept like the *context* in which an utterance is interpreted without also countenancing a vast array of interrelated notions. In this way, it made no sense to talk about the context of utterance interpretation without addressing the *knowledge* of speakers and hearers, their *purpose* or *goal* in speaking, their pre-existing *social obligations* and much else besides. As our mining continued, we gradually became aware that we were embarked on a descriptive process which had no end in sight. Nevertheless, this was a process which revealed valuable insights into the nature of the rational and other processes by means of which utterance interpretation proceeds. Moreover, this descriptive process revealed those insights without the slightest pretension of being a cognitive scientific *theory* of utterance interpretation. It is this very same process of description which I now urge pragmatists to adopt as they pursue their many and varied explorations of utterance interpretation.

### Conflict of interest statement

The author declares that the research was conducted in the absence of any commercial or financial relationships that could be construed as a potential conflict of interest.

## Footnotes

^1^These proposals include most notably contributions from relevance theory (Wilson and Sperber, 1991; Sperber and Wilson, 2002; Sperber, 2005; Wilson, 2005) and modular pragmatics theory (Kasher, 1991a,b, 1994).

^2^The term “primary pragmatic processes” is used by Recanati (1993) to refer to pragmatic developments of logical form. These processes include saturation, free enrichment, and transfer. The reader is referred to Bezuidenhout (2010) for an excellent discussion of these processes.

^3^For a detailed examination of this evidence, the reader is referred to chapter 2 in Cummings (2014a).

^4^It may be objected that linguistic context is distinct from epistemic context and that, for this reason, these tasks cannot reveal anything about the background knowledge that we bring to utterance interpretation. However, this knowledge should be interpreted broadly to refer to any information that we may use to interpret utterances. All information, including information from the linguistic context of an utterance, is background knowledge in this sense.

^5^The reader is referred to Cummings (2012b) and chapter 4 in Cummings (2005a) for discussion of those insights such as they relate to utterance interpretation.
